# APOE‐ ε4, social determinants of health and Alzheimer's Disease pathology and dementia: findings from the Biobank for Aging Studies

**DOI:** 10.1002/alz70860_105811

**Published:** 2025-12-23

**Authors:** Naomi Vidal‐Ferreira, Lea T. Grinberg, Aline Maria Macagnan Ciciliati, Eduardo Ferriolli, Vitor Ribeiro Paes, Carlos Augusto Pasqualucci, Renata Elaine Paraizo Leite, Claudia Kimie Suemoto

**Affiliations:** ^1^ Amazonia Adventist College, Benevides, PA, Brazil; ^2^ University of Sao Paulo Medical School, Sao Paulo, Brazil; ^3^ Global Brain Health Institute, University of California, San Francisco, San Francisco, CA, USA; ^4^ Memory and Aging Center, UCSF Weill Institute for Neurosciences, University of California, San Francisco, San Francisco, CA, USA; ^5^ University of São Paulo Medical School, São Paulo, São Paulo, Brazil; ^6^ University of Sao Paulo Medical School, São Paulo, Brazil; ^7^ Physiopathology in Aging Laboratory (LIM‐22), University of Sao Paulo Medical School, São Paulo, São Paulo, Brazil; ^8^ University of São Paulo Medical School, São Paulo, Brazil; ^9^ Biobank for Aging Studies of the University of São Paulo Medical School, Brazil, São Paulo, Brazil; ^10^ Division of Geriatrics, Department of Internal Medicine, University of São Paulo Medical School, São Paulo, São Paulo, Brazil; ^11^ Biobank for Aging Studies of the University of São Paulo, São Paulo, São Paulo, Brazil

## Abstract

**Background:**

APOE‐ε4 carriers and individuals with unfavorable social determinants of health (SDH) profiles have an increased risk for Alzheimer's Disease (AD). However, the modification effect of SDH on the associations of APOE‐ε4 with AD pathology or AD pathology with AD dementia symptoms is yet to be understood, particularly in low‐ to middle‐income countries where social disparities play an important role in AD burden and might interact with genetic variants.

**Method:**

This cross‐sectional study used data from the Biobank for Aging Studies. Individuals aged 50 years or older and whose next of kin (NOK) had at least a weekly contact with the deceased were included. Other causes of dementia besides AD pathology were excluded (*n* = 196). Individuals were classified into APOE‐ε4 carriers (at least one ε4 allele) and non‐carriers. The individual's NOK provided information on SDH. AD neuropathological changes (ADNC) were evaluated following international criteria using the CERAD and Braak stagings. Clinical Dementia Rating ‐ Sum of Boxes (CDR‐SB) was used to assess the extent of AD symptoms. Confirmatory factor analysis was conducted to create an SDH general factor. Logistic and linear regressions were used to investigate the associations of APOE‐ε4 with AD pathology and AD pathology with AD symptoms, respectively. Interactions of the SDH factor with APOE‐ε4 and AD pathology were tested for both associations. SDH‐profile‐stratified (favorable vs unfavorable SDH profiles based on the SDH factor's median) analyses were conducted for significant interactions.

**Result:**

In 1,021 individuals, the mean (SD) age was 74.2(12.6) years old, 51.2% were women, and 35.1% were Black/Brown. The association of APOE‐ε4 genotype with AD pathology was not modified by the SDH factor (*p* = 0.264), while the association of AD pathology with AD symptoms was modified by the SDH factor (*p* = 0.001). In stratified analysis, the association of AD pathology with AD symptoms was stronger in the unfavorable (β=8.42; 95%CI=7.44, 9.39; *p* <0.001) compared to the favorable SDH group (β=7.90; 95%CI=7.04, 8.77; *p* <0.001).

**Conclusion:**

SDH did not modify the association between APOE‐ε4 and AD pathology, while it modified the expression of dementia in participants with AD pathology. This association was stronger in individuals with unfavorable compared to favorable SDH profile.

